# A Circadian Clock Gene, *Cry*, Affects Heart Morphogenesis and Function in *Drosophila* as Revealed by Optical Coherence Microscopy

**DOI:** 10.1371/journal.pone.0137236

**Published:** 2015-09-08

**Authors:** Aneesh Alex, Airong Li, Xianxu Zeng, Rebecca E. Tate, Mary L. McKee, Diane E. Capen, Zhan Zhang, Rudolph E. Tanzi, Chao Zhou

**Affiliations:** 1 Department of Electrical and Computer Engineering, Lehigh University, Bethlehem, PA, United States of America, 18015; 2 Center for Photonics and Nanoelectronics, Lehigh University, Bethlehem, PA, United States of America, 18015; 3 Genetics and Aging Research Unit, Department of Neurology, Massachusetts General Hospital and Harvard Medical School, Boston, MA, United States of America, 02129; 4 Department of Pathology, The 3rd Affiliated Hospital of Zhengzhou University, Zhengzhou, Henan, P.R. China, 450000; 5 Program in Membrane Biology, Massachusetts General Hospital and Harvard Medical School, Boston, MA, United States of America, 02115; 6 Bioengineering Program, Lehigh University, Bethlehem, PA, United States of America, 18015; University of Valencia, SPAIN

## Abstract

Circadian rhythms are endogenous, entrainable oscillations of physical, mental and behavioural processes in response to local environmental cues such as daylight, which are present in the living beings, including humans. Circadian rhythms have been related to cardiovascular function and pathology. However, the role that circadian clock genes play in heart development and function in a whole animal *in vivo* are poorly understood. The *Drosophila* cryptochrome *(dCry)* is a circadian clock gene that encodes a major component of the circadian clock negative feedback loop. Compared to the embryonic stage, the relative expression levels of *dCry* showed a significant increase (>100-fold) in *Drosophila* during the pupa and adult stages. In this study, we utilized an ultrahigh resolution optical coherence microscopy (OCM) system to perform non-invasive and longitudinal analysis of functional and morphological changes in the *Drosophila* heart throughout its post-embryonic lifecycle for the first time. The *Drosophila* heart exhibited major morphological and functional alterations during its development. Notably, heart rate (HR) and cardiac activity period (CAP) of *Drosophila* showed significant variations during the pupa stage, when heart remodeling took place. From the M-mode (2D + time) OCM images, cardiac structural and functional parameters of *Drosophila* at different developmental stages were quantitatively determined. In order to study the functional role of *dCry* on *Drosophila* heart development, we silenced *dCry* by RNAi in the *Drosophila* heart and mesoderm, and quantitatively measured heart morphology and function in those flies throughout its development. Silencing of *dCry* resulted in slower HR, reduced CAP, smaller heart chamber size, pupal lethality and disrupted posterior segmentation that was related to increased expression of a posterior compartment protein, wingless. Collectively, our studies provided novel evidence that the circadian clock gene, *dCry*, plays an essential role in heart morphogenesis and function.

## Introduction

Circadian rhythms recur regularly over an approximately 24-hour cycle affecting many aspects of biological processes in the living beings ranging from tiny microbes to higher order animals including humans. Circadian rhythms are important in determining the cellular and tissue processes as well as whole-body functions such as sleep and other biological activities linked to this daily cycle [1]. Circadian rhythms are controlled by circadian clock genes that form the positive and negative feedback loops. The core negative feedback loop of the circadian clock is compromised of the circadian locomotor output cycles kaput (*Clock*), the brain and muscle aryl hydrocarbon receptor nuclear translocator-like protein (*Bmal1*), the period (*Per*) and the cryptochrome (*Cry*) genes. The negative feedback loop is built by a cyclic process in which the heterodimeric *Clock*/*Bmal1* complex drives the expression of its own inhibitors, the *Per* and the *Cry* [2–6]. As described previously, the *Per* dimerizes with the *Cry* to form the negative element of the feedback loop that translocates into the nucleus and inhibits the activity of *Clock/Bmal1* complex [7]. The repression of the *Clock/Bmal1* complex is relieved when the *Per/Cry* complex is degraded, and the circadian cycle begins again [2–7].

Studies have shown that circadian rhythms are related to cardiovascular function and pathology [8]. Heart rate (HR) exhibits variations consistent with circadian rhythms [8]. Several cardiovascular disorders including myocardial ischemia, acute myocardial infarction, sudden cardiac death and cardiac arrhythmias show clear temporal patterns related to the circadian rhythms. However, genes involved in the circadian regulation of the heart development and their underlying pathways are poorly understood.

*Drosophila melanogaster*, commonly known as the fruit fly, has been widely used as a model organism in developmental biology and genetic research. The *Drosophila* heart shares many similarities with vertebrates in heart development, underlying genetic mechanisms and functional pathways regulating circadian rhythms and cardiac function [8–11]. The *Drosophila* cryptochrome (*dCry*) gene encodes a major component of the circadian clock and regulates circadian rhythm in *Drosophila* by acting on an evolutionarily well conserved circadian negative feedback loop [7, 12–14]. *dCry* is autonomously expressed in multiple internal organs, including the heart [15]. Flies with a *cry* ^*b*^ null mutation in the *dCry* exhibit weakness, poor synchronization to light dark cycles, external blindness, and taking several hours for daily rhythm resets. These *cry* ^*b*^ flies show no response to brief light pulses. These findings indicate that *Cry* regulates the light-mediated entrainment and the daily rhythms of locomotion [14, 16]. In addition, both *cry* ^*b*^ and another null mutant, cry^02^, flies fail to respond to magnetic field [17], which suggest that *Cry* functions in response to magnetic field. To our knowledge, the role of circadian clock genes, such as *dCry*, in heart development and function throughout *Drosophila*’s lifecycle has not yet been investigated in a whole animal *in vivo*.

Optical coherence tomography (OCT) is an *in vivo* three-dimensional (3D) imaging technique, which can provide images of biological tissues in real-time with micron-scale spatial resolutions and a penetration depth of 1–2 mm [18–20]. OCT has been successfully used to assess the structure and function of the developing heart in various animal models [21–24]. Previous studies in adult *Drosophila* using OCT were able to quantitatively characterize functional parameters of its heart *in vivo* and could determine the functional roles of various candidate genes in cardiac functioning [25–28]. Optical coherence microscopy (OCM) is an extension of OCT combining the advantages of both OCT and confocal microscopy. OCM enhances the optical sectioning performance and provides higher transverse resolution using high numerical aperture objectives [29–31]. *Drosophila*’s heart tube is located on the dorsal side of its abdomen, usually within 200 μm from the tissue surface. Hence, it is possible to analyse structure and function of the *Drosophila* heart at different developmental stages using OCM.

In this study, we utilized a high speed and ultrahigh resolution OCM system for 3D and M-mode (2D + time) imaging of the *Drosophila* heart with micron-scale resolutions. For the first time, we performed longitudinal analysis of functional and morphological changes in the *Drosophila* heart of the same specimen throughout its post-embryonic lifecycle. We silenced *dCry* by RNAi in the *Drosophila* heart and mesoderm, and quantitatively characterized the functional effect of *dCry* in the heart throughout its development. Multiple signalling pathways regulate the *Drosophila* wing development, such as the Wnt signalling pathway which is critical in both wing development and heart formation. We therefore used the *Drosophila* wing pattern as an important tool for characterizing the role of *dCry* in Wnt signalling pathway.

## Results

### Longitudinal volumetric and functional imaging of the *Drosophila* heart development using OCM

**Fig 1:**
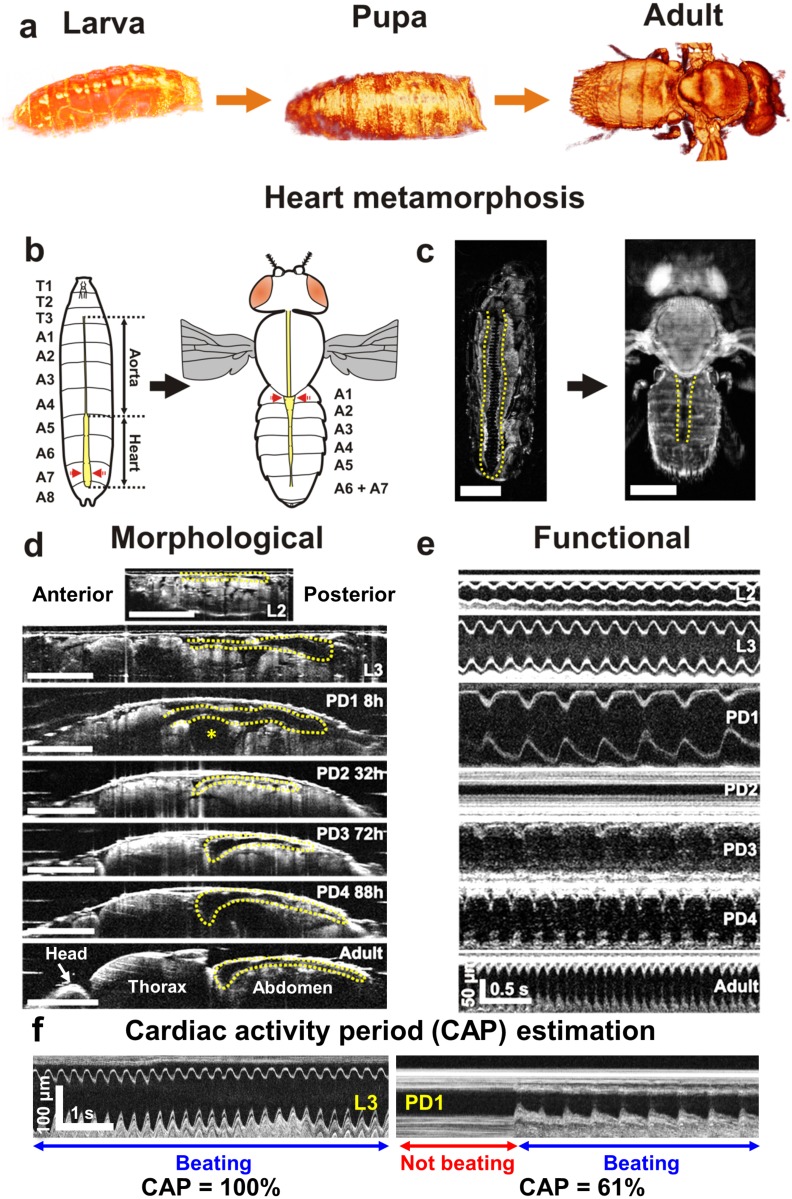
3D and M-mode OCM imaging of post-embryonic *Drosophila* lifecycle. (**a**) 3D OCM renderings of a 24B-GAL4/+ *Drosophila* flies at larva, pupa and adult stages. (**b**) Schematic representation of heart metamorphosis. Red arrows on larva and adult schematic denote the OCM M-mode imaging locations until PD1 24h and for subsequent time points, respectively. (**c**) Enface OCM projections showing heart metamorphosis. (**d**) Axial OCM sections showing heart remodelling during Drosophila lifecycle. * denotes the air bubble location during early hours of pupa development. (**e**) M-mode images at different developmental stages showing HR changes across lifecycle. (**f**) Examples demonstrating cardiac activity period (CAP) calculation. Scale bars in (c) and (d) represent 500 μm.

We analysed the structural and functional changes of the *Drosophila* heart throughout its post-embryonic lifecycle, including larva stage 2 (L2) and stage 3 (L3), pupa day 1, 2, 3, 4 (PD1, PD2, PD3, PD4) and adult day 1 (AD1) to monitor the cardiac development at different developmental stages using OCM. Fig 1a shows 3D OCM renderings of a 24B-GAL4/+ *Drosophila* specimen at larva, pupa and adult stages, respectively. The *Drosophila* heart exhibited significant structural remodelling during metamorphosis to become an adult heart (Fig 1b and 1c). The larval heart extends from the last abdominal segment (A8) to the dorsal anterior region of the brain [32]. The posterior heart region has a broader lumen between the abdominal segments A5–A8. The anterior portion between segments T3–A5, termed as aorta, has a narrower diameter. In the L2 stage, the heart tube was dorsally and medially located, and grew bigger in size during the L3 stage. In agreement with previous observations [33], a moving air bubble appeared inside the pupa during the early hours of PD1 (* in Fig 1d). The heart tube ran axially over the top of the air bubble. Subsequently, bubble disappeared in a couple of hours (~ 10–13 h) after puparium formation (APF) and imaginal head sac was everted. Since the anterior portion of the heart tube was ventrally located during this time, the entire length of heart tube except the posterior region was not visible in the OCM images ~ 12 h APF. During the second half of PD1, the abdominal portion of the heart tube (A1–A8) migrated upwards to line up with the dorsal abdomen (S1 Fig). Similar to previous observations, the dorsal vessel aligned itself along the dorsal abdomen and the posterior region of the heart (A6–A8) was eliminated through histolysis by ~ 32 h APF [10, 34]. The conical chamber of the adult heart started to develop during PD2 and it progressively enlarged during PD3. The heart of a pharate adult during PD4 appeared very similar to that of an adult heart. In addition to the structural changes, the *Drosophila* heart manifested significant functional changes during cardiac remodelling (Fig 1e). From the transverse M-mode OCM images, we observed that the HR reduced significantly as the specimen entered the pupa stage, the heart stopped beating completely during most of PD2, resumed beating during PD3 and HR increased towards the adult stage (S1–S7 videos). Notably, the *Drosophila* heart stopped beating occasionally during the pupa stages. In order to quantify this interesting phenomenon, we estimated a new parameter termed as cardiac activity period (CAP). As can be seen from the PD1 image in Fig 1f, the heart was not beating for the first 4.8 s (off-period) and then started beating regularly for next 7.6 s (on-period). The CAP was estimated as the ratio of on-period to the total imaging time. In this case, CAP was determined to be 61% for PD1. Meanwhile, the CAP was 100% for the L3 image shown in Fig 1f as the heart did not stop beating during the entire imaging time. Similarly, CAP at different developmental stages were quantified and compared.

### Quantitative analysis of *Drosophila* heart morphology and function during development

**Fig 2:**
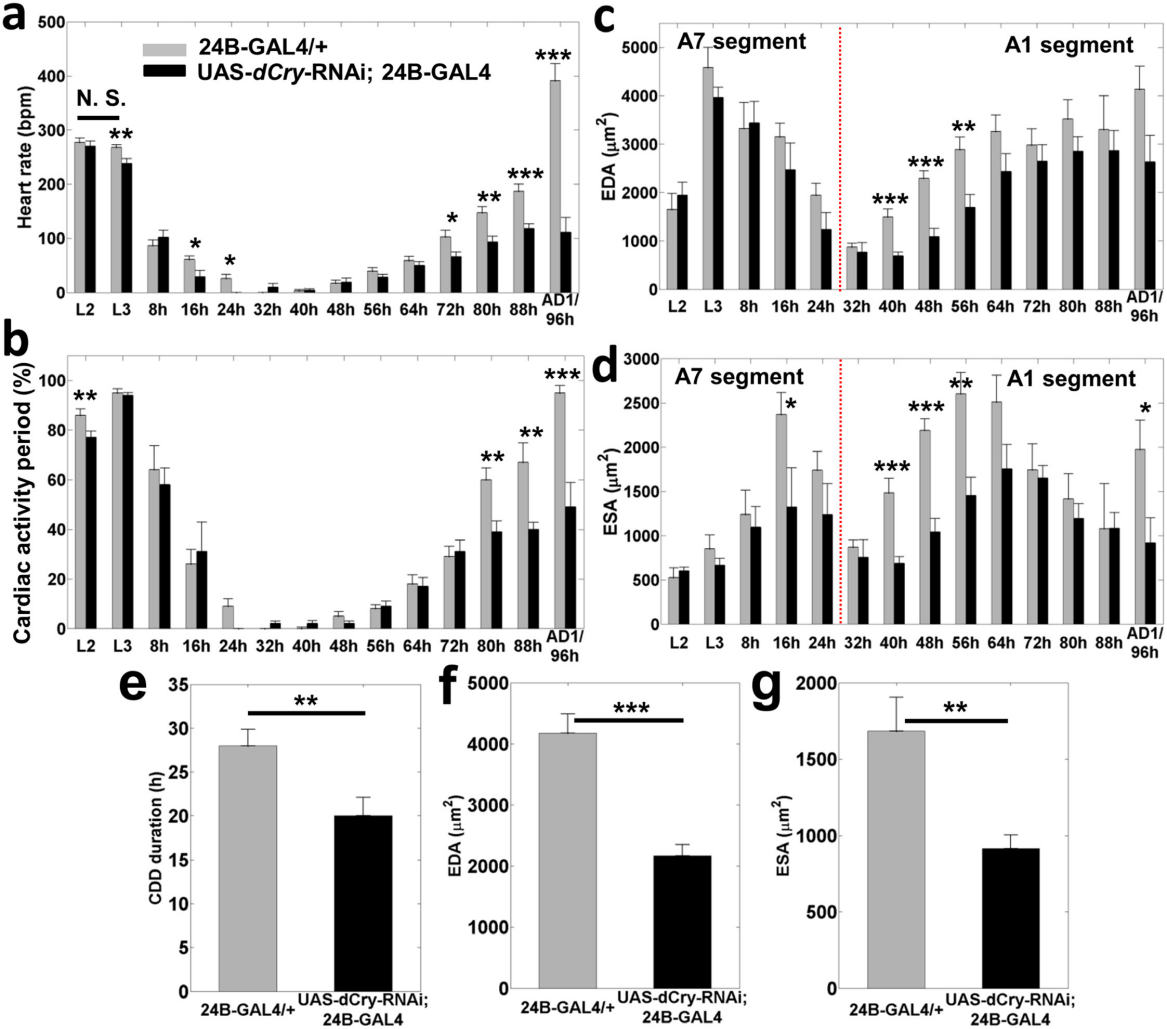
Quantitative analysis of functional and structural cardiac parameters in 24B-GAL4/+ and UAS-dCry-RNAi; 24B-GAL4 flies at different developmental stages (a-d). (**a**) Heart rate (HR), (**b**) Cardiac activity period (CAP), (**c**) End diastolic area (EDA) and (**d**) End systolic area (ESA). Both groups exhibit similar variations in HR and CAP at most of the early time points; however, differences in functional parameters became more prominent towards late pupa stages. Differences in structural parameters were more significant shortly after heart remodelling, i.e. PD2 40h, PD2 48h and PD3 56h. Red dotted line in **(c)** and **(d)** separates measurements obtained from A7 segment during early stages and those obtained from A1 segment during later stages. Black line in (**a**) represents lack of significant difference in HR between L2 and L3 (p = 0.37); all other time points showed significant difference compared to L2 (p < 0.001). * denote significant difference between 24B-GAL4/+ and UAS-dCry-RNAi; 24B-GAL4 flies at respective time points (*, p < 0.05; **, p < 0.01 and ***, p < 0.001). (**e**) Cardiac developmental diastasis duration. Comparison of (**f**) EDA and (**g**) ESA of 24B-GAL4/+ (n = 25) and UAS-dCry-RNAi; 24B-GAL4 (n = 23) flies that emerged as adult flies on adult day 1. Both EDA and ESA were significantly smaller in UAS-dCry-RNAi; 24B-GAL4 flies compared to control flies on adult day 1. Results are shown as mean ± s.e.m.

Cardiac functional and structural parameters were calculated from the transverse M-mode OCM images obtained at different developmental stages (Fig 2a–2d). In the control group (24B-GAL4/+), HR reduced from 277 +/- 8 beats per minute (bpm) at L2 stage to 26 +/- 7 bpm by the end of PD1. The *Drosophila* heart did not beat during most of PD2, while most of the heart remodelling took place. We refer to this critical and prolonged period of cardiac pause during PD2 as ‘cardiac developmental diastasis’ (CDD). The heart resumed beating at 17 +/- 6 bpm around PD2 48h, and HR increased steadily during PD3 and PD4. Among different developmental stages, the highest HR of 391 +/- 32 bpm was observed during AD1 (S1 Table). Except between L2 and L3, all other measured time points showed significant difference in HR with respect to L2 (p < 0.001; Fig 2a). The CAP increased from L2 to L3 stages, and thereafter decreased in a steady fashion until PD1 24h. When the heart resumed beating around PD2 48h, the CAP was only 5 +/- 2%. The CAP increased consistently during PD3 and PD4, and reached 95 +/- 3% at AD1, similar to the L3 stage. The CAP was significantly different at all developmental stages with respect to L2 (p < 0.05; Fig 2b).

In addition to functional differences, cardiac structural changes during the development lifecycle were also analysed. In the *Drosophila* larva, the posterior region of the heart tube had a broader lumen extending between A5 and A8 segments. To obtain consistent measurements, M-mode OCM images were obtained from the A7 segment during the larva and early pupa stages until PD1 24h. However, the posterior region of the heart was histolyzed by PD2 32h and a conical chamber started to develop *de novo* around A1 –A4 segments. Hence, M-mode images were obtained from the A1 segment for subsequent time points. End diastolic diameter (EDD), end systolic diameter (ESD) in both transverse and vertical dimensions, and hence end diastolic area (EDA) and end systolic area (ESA) of the heart increased during L2 to L3 transition (Fig 2c and 2d; S2 and S3 Figs). EDA gradually decreased alongside the reduction in HR during PD1 and the A6–A8 segments were eliminated through programmed cell death by PD2 32h (Fig 2c). EDA and ESA increased rapidly during PD2 and PD3 until 56h APF (Fig 2c and 2d). During subsequent time points until eclosion, EDA did not show large variations. However, ESA decreased gradually during this time period, indicating an increase in heartbeat amplitude. The relative changes in other structural parameters are shown in S3 Fig and S1 Table. As observed in previous studies, both the functional and structural cardiac parameters of *Drosophila* exhibited relatively high standard deviation at different developmental stages [25–27]. The primary contribution to the large standard deviation of various cardiac parameters was the natural inter-specimen variation in *Drosophila*. Secondly, even small variations in the timing of fly development can influence the measurements as the fly heart went through major functional and structural changes within a short span of time, especially during the pupa stages.

### A circadian clock gene, *dCry*, regulates heart morphogenesis and function during development

We analysed the expression of *dCry* throughout the *Drosophila* lifecycle using the quality-controlled, processed and assembled *dCry* transcriptome RNA-Seq data of whole flies from the modENCODE Project [35]. The expression of *dCry* by RNA-Seq reads was low at embryo and larva stages, but demonstrated a >100-fold increase in relative RNA expression throughout the entire pupa period and further increased to a 634-fold in AD1 compared to that at the end of embryo stage (Embryo 24 hours). In contrast, the house-keeping gene *dActin5C* demonstrated much higher RNA-Seq reads as a steady expression throughout the development (fold change <1; S2 Table). To identify whether the circadian gene *dCry* expression affects the heart development in *Drosophila*, we silenced *dCry* by RNAi specifically in heart and mesoderm with *24B-GAL4* by UAS-GAL4 system (UAS-dCry-RNAi; 24B-GAL4 abbreviated as ‘dCry-RNAi’) [36] and determined the resulting effect on heart morphogenesis and development. In order to assess the expression level of *dCry* in heart, the *dCry* was amplified by real-time RT-PCR using cDNA from the third instar larval hearts of the dCry-RNAi and the control (24B-GAL4/+). Silencing of *dCry* decreased the *dCry* expression to ~60% in dCry-RNAi larva heart compared to control larva heart. A vast majority (~98%) of the dCry-RNAi flies died during the late pupa stage and only ~2% (28/1235) of the flies survived up to the adult stage, which died within 1–3 days after eclosion.

Cardiac functional and structural parameters of the dCry-RNAi flies (n = 23) followed a similar trend as control flies (n = 21) at different time points (Fig 2a–2d). However, HR of the dCry-RNAi flies was significantly lower than controls during early developmental stages such as L3, PD1 16h and PD1 24h (p < 0.05), and the CAP was significantly lower during L2 stage (p < 0.01). Also, the CDD duration was significantly shorter in the dCry-RNAi flies (p < 0.01, Fig 2e). The reduction of HR in the dCry-RNAi flies compared to controls became more significant towards late pupa stages such as PD3 72h (p < 0.05), PD4 80h (p < 0.01) and PD4 88h (p < 0.001). Similarly, the CAP of the dCry-RNAi flies was also significantly lower at PD4 80h (p < 0.01) and PD4 88h (p < 0.01). Since most of the dCry-RNAi flies did not emerge as adult flies, OCM images were continued to be taken every 8 hours until those flies were confirmed dead (*i*.*e*. heart stopped beating). Both HR (p < 0.001) and CAP (p < 0.001) of the dCry-RNAi flies at PD4 96h were significantly lower compared to that of AD1 controls. Most of the imaged dCry-RNAi flies (10/15) died inside pupal case by PD5 104h and the rest of them were confirmed dead by PD5 120h. Unlike functional parameters such as HR and CAP that showed more prominent differences towards late pupa stages, the axial and transverse heart tube dimensions of the dCry-RNAi flies were significantly smaller compared to controls at developmental time points shortly after heart remodelling (i.e. during PD2 40h, PD2 48h and PD3 56h, p < 0.01; Fig 2c and 2d, S3 Fig and S1 Table).

**Fig 3:**
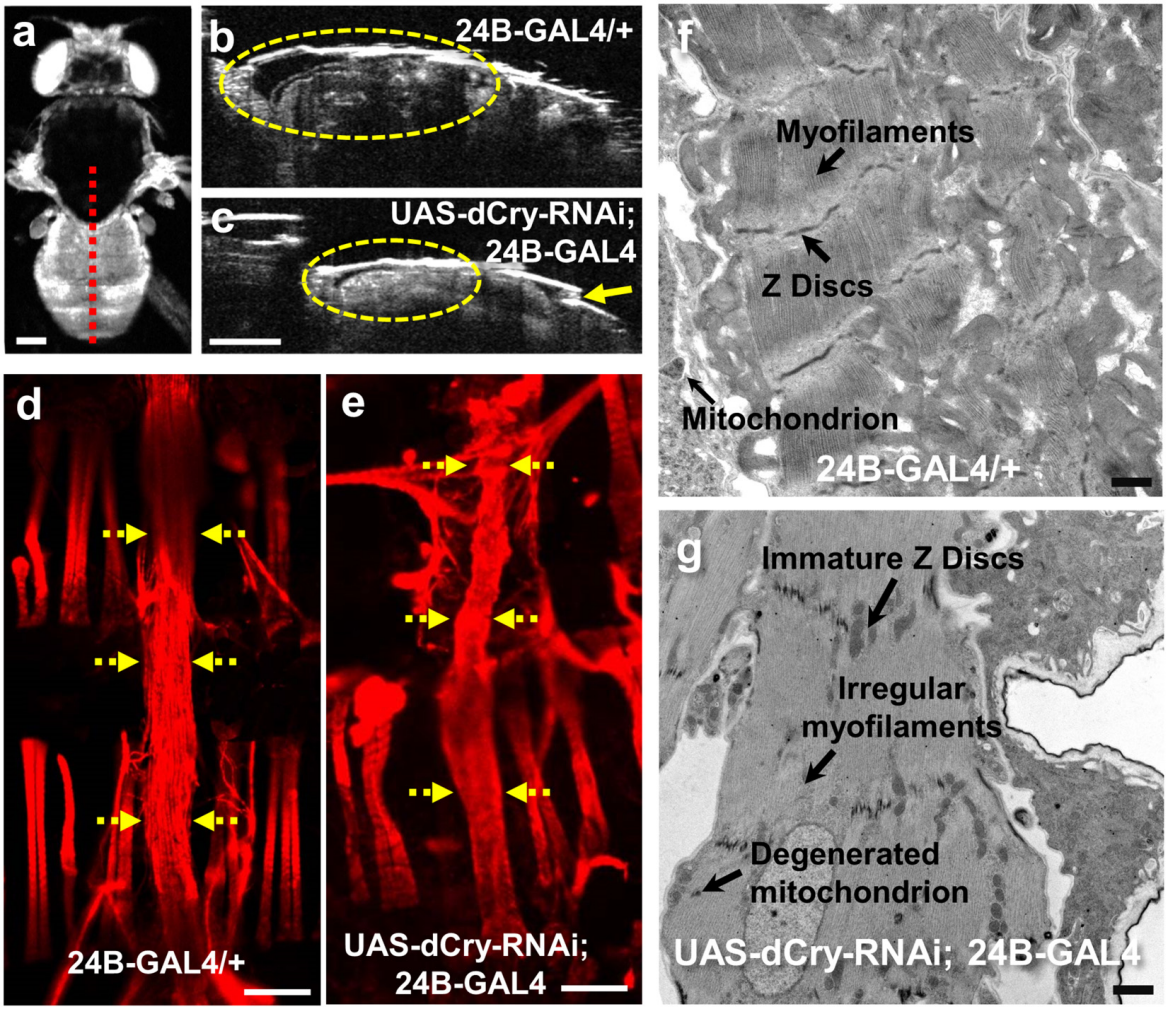
Validation of the OCM observations indicating cardiac dysfunction and structural defects in UAS-dCry-RNAi; 24B-GAL4 flies through whole heart and cardiac ultrastructure analysis. **a**) *En face* OCM projection of an adult UAS-dCry-RNAi; 24B-GAL4 fly. The red dotted line shows the imaging location corresponding to the axial OCM image shown in (**c**). (**b**, **c**) Representative axial OCM images of adult heart: (**b)** the cardiac tube of a control fly (24B-GAL4/+) appeared normal; however, (**c**) the cardiac tube of a UAS-dCry-RNAi; 24B-GAL4 fly on AD1 appeared slightly deformed with under-developed conical chamber (dotted circle) and folds along the dorsal surface (arrow). (**d, e**) Micrographs of whole adult heart by F-actin immuno-fluorescent staining: (**d)** Control fly (24B-GAL4/+) showed a normal cardiac tube. **(e)** Silencing of *dCry* in heart (UAS-dCry-RNAi; 24B-GAL4) resulted in a smaller, underdeveloped and irregular cardiac tube. Yellow dotted arrows point towards the cardiac tube. (**f, g**) Ultrastructure of adult heart longitudinal sections between A1 to A3 segments by TEM: (**f)** Control fly showed normal myofibril structure. (**g)** Silencing of *dCry* in heart resulted in immature and discontinuous Z discs, immature and irregular myofilament arrays and degenerated mitochondria. Scale bars: 200 μm in (**a**), (**b**) and (**c**), 100 μm in (**d**) and (**e**), and 500 nm in (**f**) and (**g**).

The cardiac structural and functional parameters of the few dCry-RNAi flies that emerged as adult flies (n = 23) were compared to those of age-matched control flies (n = 25) on AD1. EDA and ESA were significantly smaller (p < 0.01) in dCry-RNAi flies (Fig 2f and 2g; see S3 Table for comparison of other parameters). Underdeveloped conical chamber and folds along the dorsal abdomen were clearly observed in dCry-RNAi flies at AD1 compared to controls (Fig 3b and 3c). Fluorescent labelling of the adult heart on AD1 with F-actin immunostaining confirmed that silencing of *dCry* led to an underdeveloped, irregular and smaller cardiac tube compared to controls (Fig 3d and 3e). Transmission electron microscopy (TEM) imaging showed that adult hearts of control flies exhibited normal myofibril structure, regular myofilament arrays with continuous Z discs, normal mitochondria (Fig 3f). In contrast, hearts from the dCry-RNAi flies revealed immature and discontinuous Z discs, immature and irregular myofilament arrays and degenerated mitochondria (Fig 3g). This TEM ultrastructural analysis demonstrated that many cellular structural elements needed for normal cardiac functioning were not properly developed in the dCry-RNAi flies, even in the ones that managed to emerge as adult flies and indicates the critical role played by *dCry* in the structural development of cardiac tissue at a cellular/sub-cellular level.

### *dCry* regulates segment polarity

**Fig 4:**
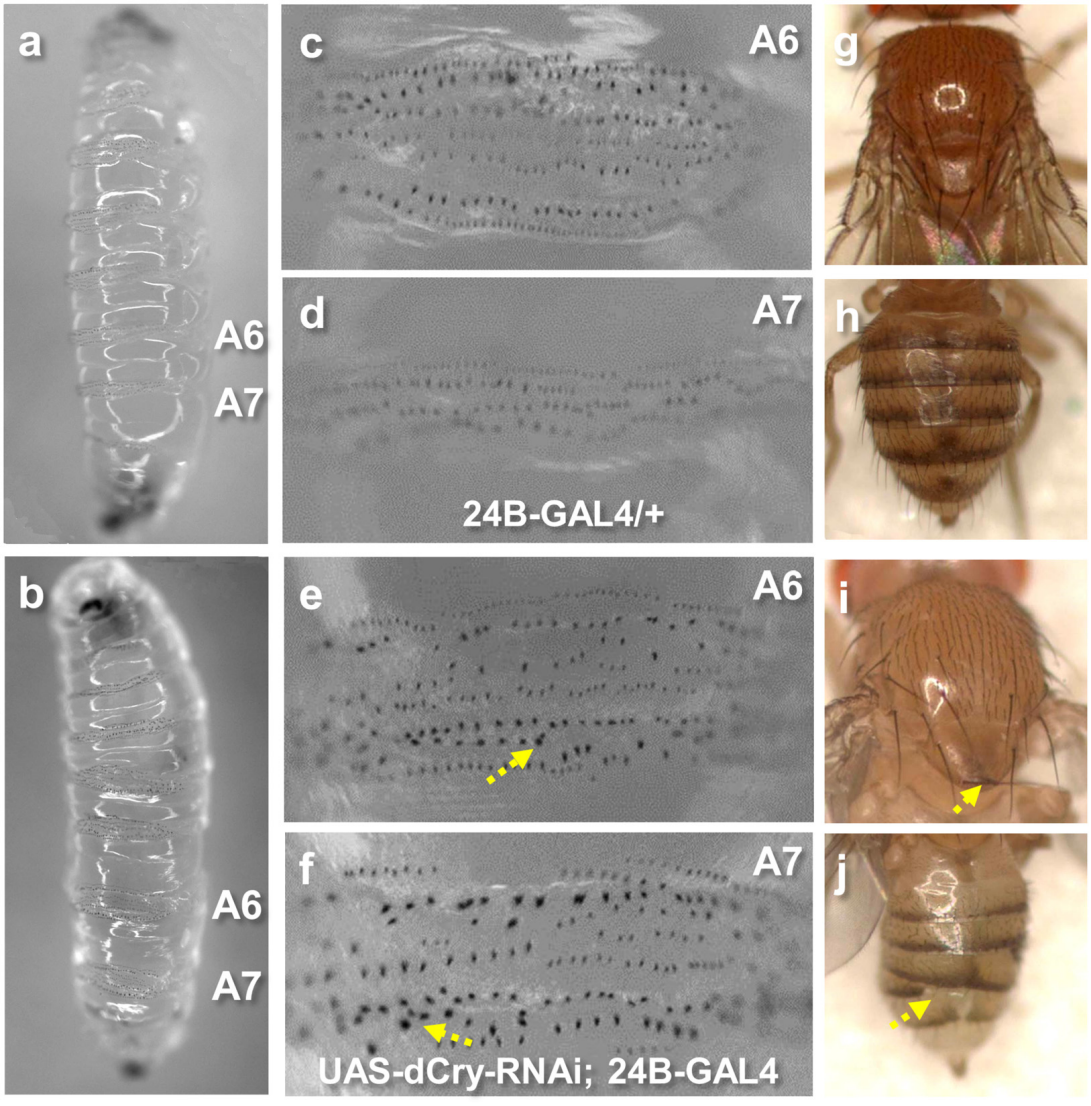
Silencing of *dCry* resulted in segment polarity phenotypes. (**a, c and d**) Control larva (24B-GAL4/+) showed regular denticle belts in posterior A6 and A7 segments. (**b, e and f**) Silencing of *dCry* (UAS-dCry-RNAi; 24B-GAL4) results in disorganized cuticular morphologies in A6 denticle belt and significantly increased, enlarged and disorganized A7 denticle belt (denoted by arrows). (**g, h**) Control flies showed normal and organized notum bristles and A6 and A7 denticle belts. (**i, j**) The few emerged UAS-dCry-RNAi; 24B-GAL4 adult flies showed a smaller notum with disoriented and up-pointing bristles in notum (arrow in **i**), and disorganized and partially absent A6 and A7 denticle belts (arrow in **j**).

In addition to the cardiac morphological and functional changes, we observed significant phenotypes related to posterior segment polarity in larva and adult flies in the dCry-RNAi group. Control third instar larva showed regular denticle belts in posterior segments (Fig 4a, 4c and 4d). In contrast, silencing of *dCry* resulted in disorganized cuticular morphologies and markedly increased, enlarged and disorganized denticle belts in the A6 and A7 segments (Fig 4b, 4e, and 4f, arrows). Consistently, the few emerged dCry-RNAi adult flies showed disorganized and partially absent denticle belts in the A6 and A7 segments (Fig 4j, arrow), compared to the well-organized A6 and A7 denticle belts in controls (Fig 4h). Moreover, the dCry-RNAi adult flies demonstrated a smaller notum with disoriented and up-pointing bristles in notum (Fig 4i, arrow) compared to the controls (Fig 4g).

**Fig 5:**
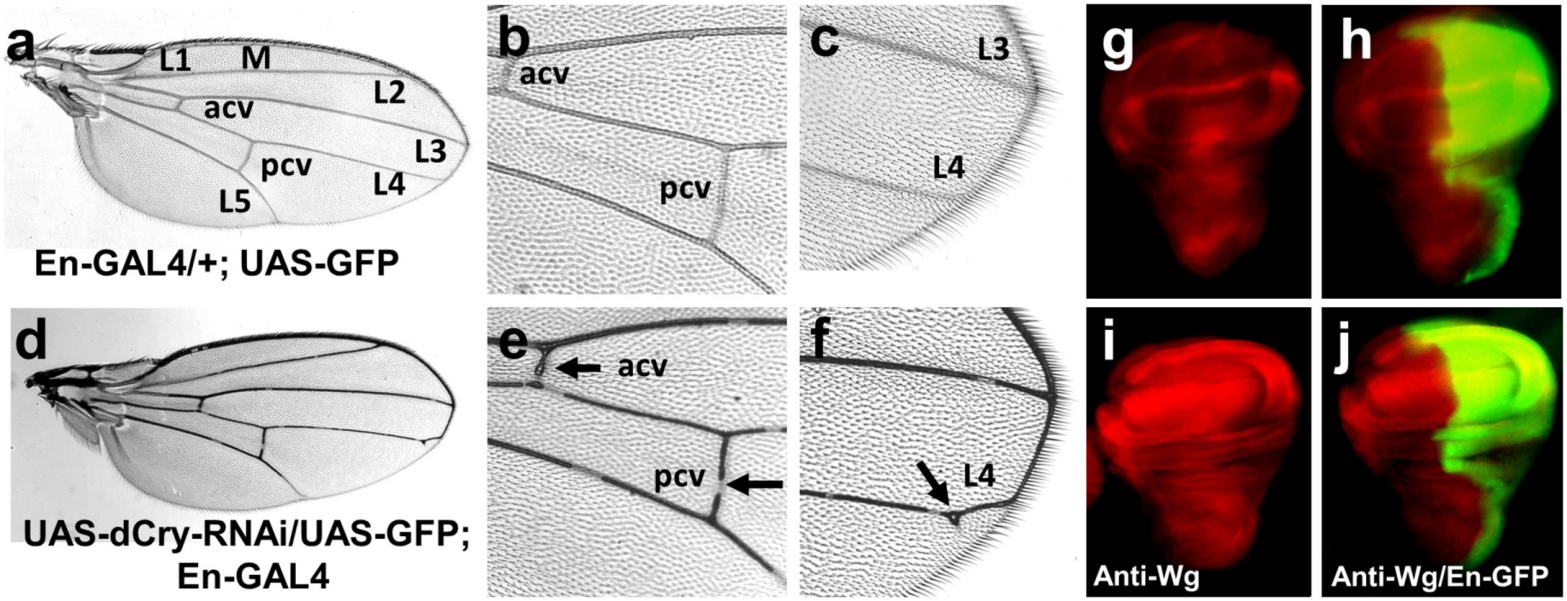
Silencing of *dCry* led to abnormal wing vein distribution and Wg expression. (**a, b and c**) Control fly with heterozygous *En-GAL4; UAS-GFP* alone (En-GAL4; UAS-GFP /+) exhibited normal wing. (**d, e and f**) Silencing of *dCry* in the wing (UAS-dCry-RNAi/UAS-GFP; En-GAL4) resulted in a marked increase in the acv, pcv (arrows in **e**), M, L3 and L4 wing veins. L4 vein was disorganized with extra veins in the distal part (arrow in **f**). (**g)** Control flies showed normal Wg expression pattern (a broad strip in the notum, a thinner strip in the prospective wing margin-dorsal/ventral (D/V) boundary, and a strip encircling the prospective wing blade). (**h)** Merged images of the expression of *Wg* and co-overexpression of GFP in the pattern of *En* in control flies. (**i)** In the dCry-RNAi wing discs, Wg expression level was markedly increased and Wg expression pattern was disorganized. (**j)** Merged images of the expression of Wg and co-overexpression of GFP in the pattern of *En* in UAS-dCry-RNAi/UAS-GFP; En-GAL4 flies.

Next, we employed the *Drosophila* wing as a tool to identify the signalling pathways that *dCry* may affect [37]. Engrailed (*En*) and inverted (*inv*) are the transcription factors that regulates the *Drosophila* posterior segmentation of the wing [38, 39] by suppressing proteins in the posterior compartments, including wingless (Wg), decapentaplegic, and patched. To identify the functional effects of *dCry* in posterior segment of the wing, we silenced *dCry* in the wing using *En-GAL4* and analysed wing phenotypes in progenies. Silencing of *dCry* by *En-GAL4* (UAS-dCry-RNAi/UAS-GFP; En-GAL4) resulted in excess anterior crossvein (acv), posterior crossvein (pcv) and longitudinal (L3 and L4) wing veins, and a disorganized posterior L4 wing vein compared to that in controls (Fig 5a–5f, arrows). We immuno-stained the third instar larva wing discs with anti-Wg antibody to check the expression of the posterior compartment protein, Wg. In the third instar wing discs of the control flies, Wg was expressed in the notum, the prospective wing margin and the prospective wing blade (Fig 5g), similar to observations in previous studies [27]. In contrast, in the dCry-RNAi wing discs, Wg expression level was increased and Wg expression pattern was disorganized (Fig 5i). GFP was co-overexpressed in both control and *dCry*-RNAi flies to demonstrate the posterior *En* expression pattern (Fig 5h and 5j). This data demonstrated that the reduced *dCry* expression increased the posterior compartment protein Wg expression.

## Discussion

Circadian rhythms play a significant role in a variety of basic physiological processes, which are controlled by the circadian clock [7]. However, the importance of the circadian clock genes in regulating heart development and function remains elusive. *dCry* plays a significant role in controlling the circadian rhythms [12–15]. In this study, we have shown that the silencing of *dCry* resulted in slower HR, reduced CAP, smaller heart chamber size at various developmental stages, pupal lethality, and larva and adult phenotypes related to segment polarity. These novel findings helped to identify the functional role played by the circadian clock gene, *dCry* in heart morphogenesis and function during development.

Several methods such as immunostaining, *in vivo* time-lapse studies using GFP-labelled specimens and high-speed video microscopic techniques have been used to investigate the *Drosophila* heart development [40–42]. Due to the lack of appropriate *in vivo* imaging tools providing sufficient resolution, speed and penetration depth, comprehensive longitudinal studies investigating both cardiac structural and functional variations across post-embryonic stages in *Drosophila* have been very limited to date. To our knowledge, this is the first study to non-invasively monitor and systematically analyse heart development in the same *Drosophila* specimen throughout its post-embryonic development lifecycle. Label-free OCM images obtained every 8 hours during the pupa stage helped us to gain a deeper understanding of the heart metamorphosis and functional changes associated with this process. Although previous studies have observed varying intermittent periods of cardiac activity during pupa stage [43, 44], quantitative assessment of HR and CAP, and the nature of their variations across the pupa stage have not been reported before. To our knowledge, the CDD duration is also quantified for the first time. The CDD period during PD2 may facilitate the morphological rearrangements of the heart, such as conical chamber formation and histolysis of the posterior portion. The gradual increase in HR and CAP during PD3 and PD4 may be necessary to meet the higher metabolic demands and to prepare the pharate adult for eclosion. The quantitative structural and functional information obtained using OCM from the same specimen at different developmental stages can be used to advance our understanding of cardiac development, and to characterize the role of specific genes and related functional pathways involved in heart formation and functioning.

Our findings that *dCry* regulates heart morphogenesis and function support the contention of a critical role of the circadian clock genes in heart development and function as indicated in the previous studies. Association has been found between circadian rhythm disruption and increased incidence of cardiovascular disorders, such as hypertension, coronary artery disease and other cardiovascular pathologies in human and animal models [45, 46]. It was shown that mice deficient for another core circadian clock gene, *Bmal1*, exhibited age-dependent dilated cardiomyopathy [46]. The core circadian clock genes affect the expression of downstream target genes like *xNocturnin* in all mammalian cells to regulate circadian rhythm-associated physiology and behaviour [7] as well as the key cardiovascular system components (*e*.*g*. cardiomyocytes and vascular smooth muscle cells) [15, 47]. In addition, the circadian clock plays an important role in chronic vascular responses [48]. Mice with either a deficient *Bmal1* or mutant *Clock* exhibited a dysfunctional circadian clock with vascular injury and endothelial dysfunction. Mice with deficient *Bmal1* also showed significantly attenuated Akt and subsequent nitric oxide signalling in arteries, which suggest that *Bmal1* is involved in a pathway critical to vascular function [48]. It has been shown that rat hearts exhibit marked rhythmicity in the expression of the core circadian clock components and output genes [49]. Moreover, the intracellular circadian clocks in the heart and vasculature ensure an optimal response to various stimuli, including sympathetic and autonomic nervous activities. Changed expression of the core circadian clock genes may impair the function of the intracellular circadian clocks, which disrupts the response of the key cardiovascular system components to environmental stimuli, resulting in the development of cardiovascular disease [15, 47]. The role of circadian genes such as *Cry* in heart development and function may explain the association between cardiovascular disorders and activity patterns related to circadian rhythms [50].

In a study analysing the rhythmic expression of four circadian clock genes (*PER1*, *PER2*, *BMAL1*, and *CRY1*) in hearts of patients with coronary heart disease, cardiomyopathy or healthy controls, *CRY1* mRNA level did not show a circadian rhythm in any of the study subjects [51]. However, expressions of *PER1*, *PER2*, and *BMAL1* mRNAs were correlated with circadian rhythms in hearts, which suggests that *CRY1* may have more function in human heart in addition to regulating the circadian clock negative feedback loop. We employed the *Drosophila* wing disc as a tool to explore the functional pathways of *dCry*. Silencing of *dCry* in the wing using *En-GAL4* resulted in disorganized and excess posterior wing phenotypes and increased posterior compartment protein Wg expression. This novel finding indicates that *dCry* may be similar to *En* as a regulator of posterior segment polarity by suppressing the posterior compartment proteins such as Wg. Wg is known to specify segmental polarity, mesoderm and heart development. Wg is a major component of Wnt signalling pathway, which is critical in heart formation [52, 53]. Flies with down-regulated Wnt pathway exhibit a under-developed cardiac tube [54]. The regulation of Wg expression by *dCry* may contribute to the molecular mechanism involved in the pathogenesis of heart tube malformation during development and cardiac dysfunction induced by silencing of *dCry*. Further studies will define the functional role and underlying functional pathways of *dCry* in the process of segmentation, such as morphogen gradients, cell-cell signalling and the origin of patterning in *Drosophila*.

In summary, we have shown that reduced expression of *dCry* by RNAi in the heart and mesoderm resulted in cardiac dysfunction, pupae lethality and disrupted posterior segmentation that were related to the increased expression of a posterior compartment protein, Wg. Together, this data provided novel *in vivo* evidence for an important functional role for a circadian clock gene, *dCry*, in heart development and cardiac function.

## Materials and Methods

### Transgenic flies and fly culture

Fly crosses were carried out according to standard procedures as described previously [27, 55]. Flies were cultured in tubes containing standard fly food. The fly strains utilized in this study included: UAS-dCry-RNAi, 24B-GAL4 [56], and En-GAL4 [38]. All the flies used in this study were exposed to a light–dark cycle of 24 h (12:12 h).

### Animal Preparation

For all the specimens imaged longitudinally using OCM, the specimens were collected at L2 and mounted on a glass slide for obtaining 3D and M-mode OCM images. After imaging, the specimens were kept in separate tubes for continuous development. The specimens were taken out for OCM imaging at L3, every 8 hours during the pupa stage and AD1 stages to monitor the cardiac development at different developmental stages. In the adult stage, flies were anesthetized and imaged as described in detail previously [27, 41]. M-mode images were obtained from the A7 segment until PD1 24h and from the A1 segment for later stages of development. In this study, over 93% of the specimens survived to the adult stage. As only 2% of the UAS-dCry-RNAi; 24B-GAL4 flies emerged as adult flies, OCM images were obtained from additional specimens on AD1, which were not measured in the longitudinal study.

### OCM system and imaging analysis

A portion of the white light spectrum from the supercontinuum source, (SC-400-4, Fianium Ltd., UK) with a centre wavelength of ~800 nm and a bandwidth of ~220 nm was used as the light source for the OCM system. Similar to ref. [57], the OCM system used a 45° rod mirror to generate an annular sample probe beam for obtaining extended depth of focus. The axial and transverse resolutions provided by the OCM system was 1.5 μm and 3.9 μm respectively. The backscattered light from the reference and sample arms was detected using a spectrometer comprising a 600 lines/mm transmission grating (Wasatch Photonics, Logan, UT, USA) and a 2048 pixel line-scan camera, (AViiVA EM4, e2v technologies plc, UK), operated at 20k A-scans/s. Sensitivity of the system was determined to be ~ 95 dB with a sample arm power of 5 mW. M-mode images were acquired at a frame rate of 128 Hz.

3D OCM images were analysed using ImageJ (National Institutes of Health, USA) to visualize the structural development of *Drosophila* heart. Transverse M-mode images (2D + time) obtained across the heart over a region covering 0.28 x 0 x 0.57 mm^3^ (128 x 800 x 2048 voxels) were analysed using a custom program written in MATLAB. The heart tube region was segmented out from the M-mode datasets using a magic wand algorithm, and systolic and diastolic locations were subsequently identified using a peak-finding algorithm. EDD and ESD represented the diameter of the heart tube at diastole and systole respectively (S2 Fig). Fractional shortening (FS) along each dimension was calculated from these values as: $FS\;=\;\frac{\left(EDD-ESD\right)}{EDD}\times 100$. Moreover, the area of the heart tube during diastole and systole (EDA and ESA) was also determined.

As *Drosophila* heart stopped beating occasionally, especially during the pupa stages, the CAP at different developmental stages were calculated. A MATLAB algorithm was developed to automatically detect the beating duration (on-period) and the resting duration (off-period) within each recording session spanning ~ 30 s. To circumvent the random nature of cardiac beating, five separate recordings of M-mode images were acquired at each time point and the average CAP at each time point was determined. Only the time during which heart was beating (on-period) was taken into account, while calculating the HR at each time point. We used the Student’s t-test for statistical analysis and results were considered statistically significant at p *<* 0.05.

### Analysis of the Drosophila heart structures by F-actin immuno-staining and transmission electron microscopy

Hearts from 15 1-day old dCry-RNAi and control adult flies were dissected for whole heart and cardiac ultrastructure analyses as described in detail previously [27, 55]. The whole adult heart tube was fluorescently labelled with F-actin and imaged as described previously [58]. For TEM, 15 hearts from 1-day old dCry-RNAi and control flies were examined as described previously [27, 55].

### Analysis of the *Drosophila* phenotypes and Wg expression

Wing phenotypes and Wg expression in wing discs were analyzed as described in detail previously [27, 55]. 30 wings from adult flies and 30 third instar larval wing discs were analyzed. The anti-bodies used in this study included: Mouse anti-wg antibody (1:1000, Developmental Studies Hybridoma Bank, Iowa City, IA) and the secondary anti-mouse Alexa 546 (1:200, Life Technologies). The *Drosophila* larva, pupa and adult flies were examined for phenotypes and imaged with a Nikon stereo microscope.

### cDNA synthesis and real-time RT-PCR

cDNA synthesis and real-time RT-PCR reactions were performed as described in detail previously [27]. The cDNAs were synthesized from total RNA extracted from the 15 pooled third instar larval hearts using SUPERSCRIPT Pre-amplification System for First-Strand cDNA Synthesis (Life Technologies). The expressions of *dCry* or the house-keeping gene *dActin5c* were quantified using real-time RT-PCR with *dCry* or *dActin5c*-specific sense and antisense primers on an iCycler (BIO-RAD). The sequences of primers are: *dCry* 5’ GCACACGGTGCAAATTATTG, 3’ GAAGCCCATGTTGTCTCCAT; RT-PCR product size 166bp; *dActin5C* 5’ TACCCCATTGAGCACGGTAT, 3’ GGTCATCTTCTCACGGTTGG; RT-PCR product size 166bp. The relative numbers of copies (%) of mRNA molecules of the expressions of *dCry* were shown after calibrated by co-amplification of *dActin5c*.

## Supporting Information

S1 Fig Heart migration towards the dorsal abdomen during the pupa stage (a-h). Only the posterior end of the heart is visible in OCM images during PD1 12h–18h, when the anterior region is located deep. The posterior portion of the heart tube (A6–A8) is histolyzed and the anterior portion aligns along the dorsal abdomen by ~ PD2 30h. Dotted curves delineate the heart tube. Scale bars denote 500 μm.
(TIF)

S2 Fig Different phases of a cardiac cycle in *Drosophila* and definition of various structural cardiac parameters. OCM images displaying a *Drosophila* larval (L3) heart during **a)** diastole and **b)** systole. **c)** A representative M-mode image showing fractional shortening of heart along vertical dimension.
(TIF)

S3 Fig Quantitative analysis of structural cardiac parameters in 24B-GAL4/+ and UAS-dCry-RNAi; 24B-GAL4 flies at different developmental stages. **a**) EDD-horizontal, **b**) EDD-vertical, **c**) ESD-horizontal, **d**) ESD-vertical, **e**) FS-horizontal, **f**) FS-vertical, and **g**) FS-area. Results are shown as mean ± s.e.m.
(TIF)

S1 Table Comparison of cardiac structural and functional parameters of 24B-GAL4/+ and UAS-dCry-RNAi-24B-GAL4 flies at different developmental stages. (DOCX)

S2 Table RNA-seq reads of *dCry* and *dAct5C* at various *Drosophila* developmental stages from the modENCODE Project Ref.[35]. (DOCX)

S3 Table Comparison of structural parameters of 24B-GAL4/+ and UAS-dCry-RNAi-24B-GAL4 flies on the first day of the adult stage (*, p < 0.05; **, p < 0.01; ***, p < 0.001). (DOCX)

S1 Video M-mode video showing *Drosophila* heart beat during larva stage 2 (L2). This video was captured around A7 segment.
(MPG)

S2 Video M-mode video showing *Drosophila* heart beat during larva stage 3 (L3). This video was captured around A7 segment.
(MPG)

S3 Video M-mode video showing *Drosophila* heart beat during early pupa stage (PD1 16h). This video was captured around A7 segment.
(MPG)

S4 Video M-mode video showing *Drosophila* heart beat during pupa day 2 (~32h after pupa formation). Heart stops to beat for most of PD2, while heart is undergoes significant structural transformations. This video was captured around A1 segment.
(MPG)

S5 Video M-mode video showing *Drosophila* heart beat during pupa day 3 (~64h after pupa formation). Heart restarts to beat during PD3. This video was captured around A1 segment.
(MPG)

S6 Video M-mode video showing *Drosophila* heart beat during pupa day 4 (~88h after pupa formation). Heart rate increases during the late pupa stages. This video was captured around A1 segment.
(MPG)

S7 Video M-mode video showing heart beat of an adult Drosophila (day 1). Heart rate reaches the maximum during the adult stage. This video was captured around A1 segment.
(MPG)
