# Supplementary figures and images for: A Circadian Clock Gene, Cry, Affects Heart Morphogenesis and Function in Drosophila as Revealed by Optical Coherence Microscopy
Source: PLoS One. 2015 Sep 8;10(9):e0137236. doi: 10.1371/journal.pone.0137236 (PMC4565115; doi:10.1371/journal.pone.0137236)

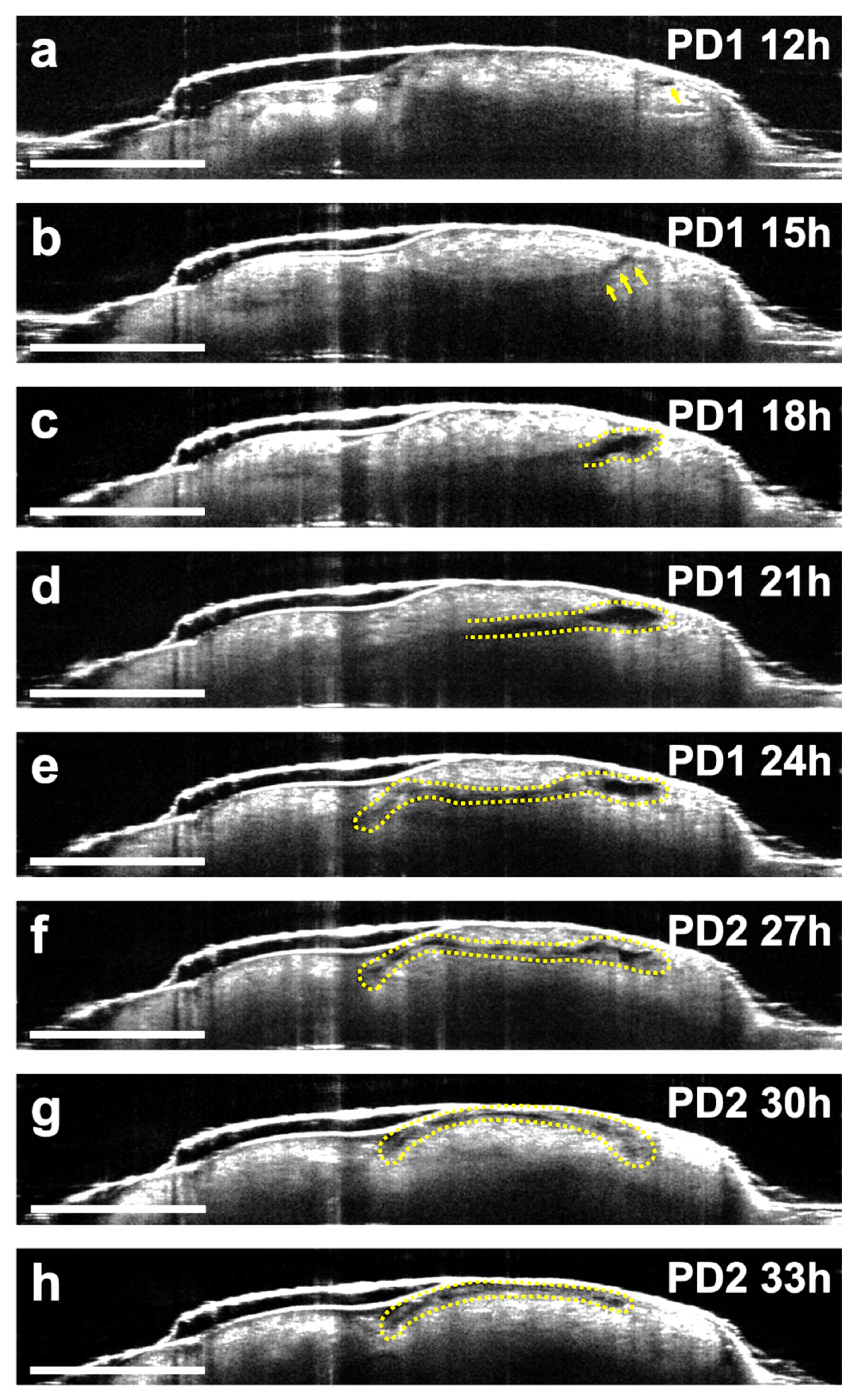

Supplement: S1 Fig — Only the posterior end of the heart is visible in OCM images during PD1 12h–18h, when the anterior region is located deep. The posterior portion of the heart tube (A6–A8) is histolyzed and the anterior portion aligns along the dorsal abdomen by ~ PD2 30h. Dotted curves delineate the heart tube. Scale bars denote 500 μm. (TIF) [file pone.0137236.s001.tif]

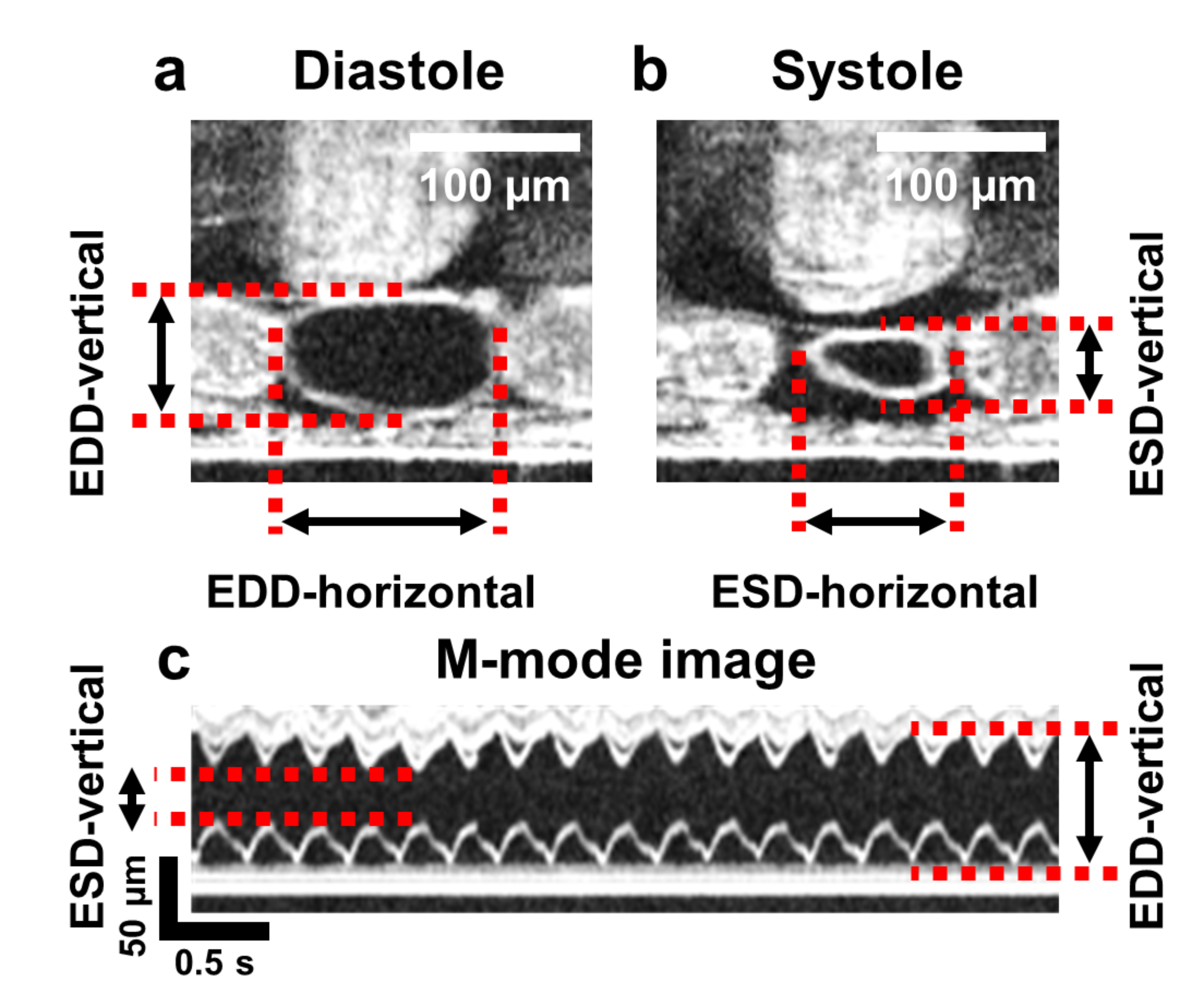

Supplement: S2 Fig — OCM images displaying a Drosophila larval (L3) heart during a) diastole and b) systole. c) A representative M-mode image showing fractional shortening of heart along vertical dimension. (TIF) [file pone.0137236.s002.tif]

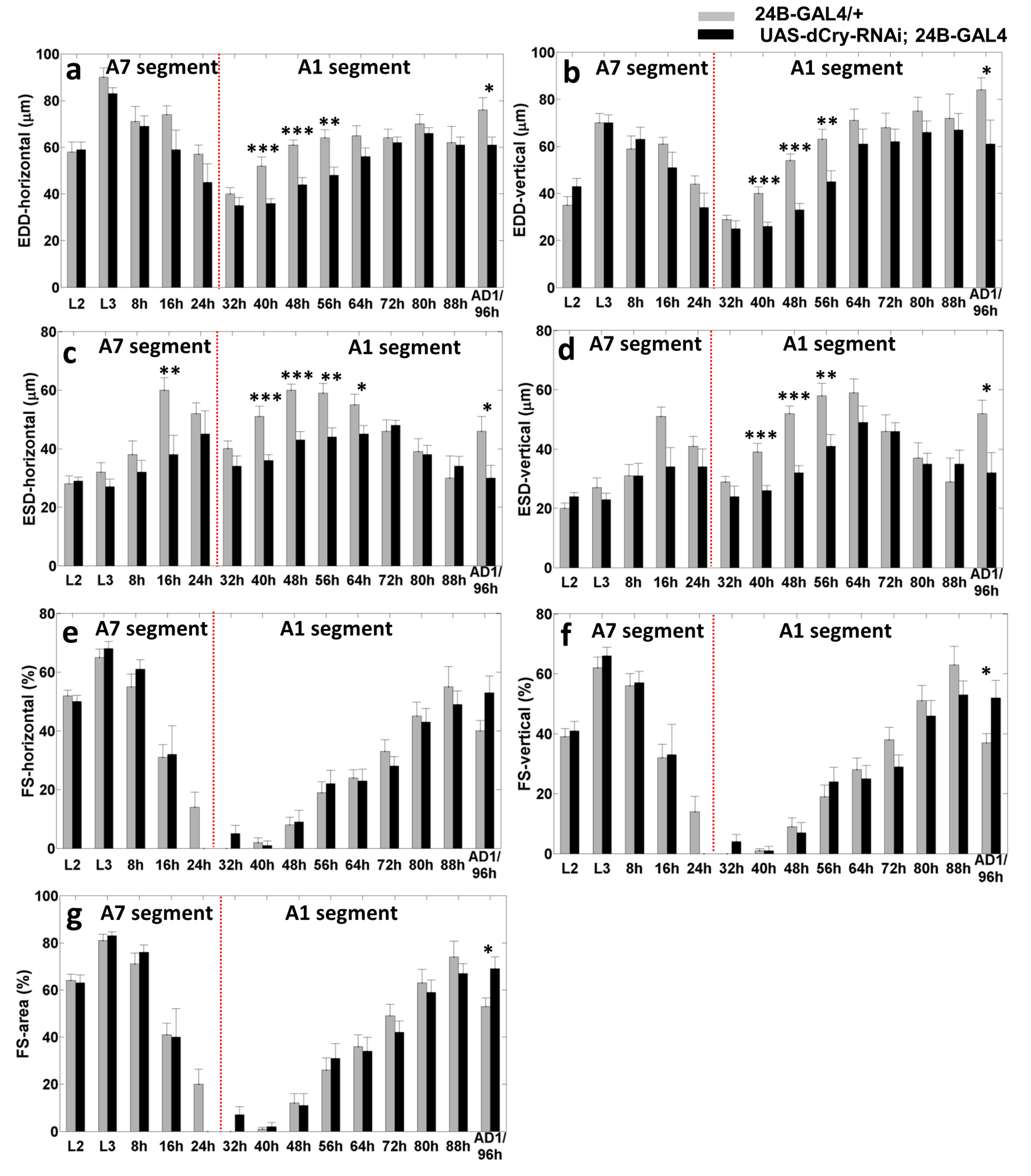

Supplement: S3 Fig — a) EDD-horizontal, b) EDD-vertical, c) ESD-horizontal, d) ESD-vertical, e) FS-horizontal, f) FS-vertical, and g) FS-area. Results are shown as mean ± s.e.m. (TIF) [file pone.0137236.s003.tif]
