# Supplementary material for: A Circadian Clock Gene, Cry, Affects Heart Morphogenesis and Function in Drosophila as Revealed by Optical Coherence Microscopy
Source: PLoS One. 2015 Sep 8;10(9):e0137236. doi: 10.1371/journal.pone.0137236 (PMC4565115; doi:10.1371/journal.pone.0137236)
Supplement: S1 Table — (DOCX) [file pone.0137236.s004.docx]

| **Cardiac parameters** | **Group** | **Developmental stage** | | | | | | | | | | | | | |
| --- | --- | --- | --- | --- | --- | --- | --- | --- | --- | --- | --- | --- | --- | --- | --- |
|  |  | **L2** (mean +/- s.e.m.) | **L3**  (mean +/- s.e.m.) | **PD1** | | | **PD2** | | | **PD3** | | | **PD4** | | **AD1/**  **96h**  (mean +/- s.e.m.) |
|  |  |  |  | **8h**  (mean +/- s.e.m.) | **16h**  (mean +/- s.e.m.) | **24h**  (mean +/- s.e.m.) | **32h**  (mean +/- s.e.m.) | **40h**  (mean +/- s.e.m.) | **48h**  (mean +/- s.e.m.) | **56h**  (mean +/- s.e.m.) | **64h**  (mean +/- s.e.m.) | **72h**  (mean +/-  s.e.m.) | **80h**  (mean +/- s.e.m.) | **88h**  (mean +/- s.e.m.) |  |
| **HR**  **(bpm)** | **24B-GAL4/+** | 277  +/- 9 | 268  +/- 5 | 86  +/- 11 | 61  +/- 6 | 26  +/- 8 | 0  +/- 0 | 3  +/- 3 | 17  +/- 6 | 39  +/- 6 | 59  +/- 7 | 103  +/- 11 | 147  +/- 13 | 187  +/- 39 | 391  +/- 32 |
|  | **n** | 21 | 17 | 13 | 19 | 19 | 19 | 19 | 19 | 19 | 18 | 17 | 18 | 9 | 14 |
|  | **dCry-RNAi** | 270  +/- 9 | 238  +/- 9 | 102  +/- 13 | 29  +/- 12 | 0  +/- 0 | 10  +/- 6 | 4  +/- 3 | 19  +/- 7 | 28  +/- 5 | 50  +/- 7 | 66  +/- 9 | 93  +/- 11 | 118  +/- 9 | 111  +/- 27 |
|  | **n** | 23 | 16 | 12 | 10 | 8 | 17 | 17 | 17 | 17 | 14 | 14 | 15 | 13 | 10 |
| **CAP (%)** | **24B-GAL4/+** | 86  +/- 2 | 95  +/- 2 | 64  +/- 10 | 26  +/- 6 | 9  +/- 3 | 0  +/- 0 | 0  +/- 1 | 5  +/- 2 | 8  +/- 1 | 18  +/- 3 | 29  +/- 4 | 60  +/- 5 | 67  +/- 8 | 95  +/- 3 |
|  | **dCry-RNAi** | 77  +/- 2 | 94  +/- 1 | 58  +/- 7 | 31  +/- 12 | 0  +/- 0 | 2  +/- 1 | 2  +/- 1 | 2  +/- 1 | 9  +/- 2 | 17  +/- 3 | 31  +/- 5 | 39  +/- 4 | 40  +/- 3 | 49  +/- 10 |
| **EDA  (µm^2^)** | **24B-GAL4/+** | 1651 +/- 327 | 4581 +/- 418 | 3319 +/- 539 | 3153 +/- 278 | 1941 +/- 246 |  | 1491 +/- 166 | 2292 +/- 153 | 2889 +/- 258 | 3257 +/- 343 | 2977  +/- 338 | 3514 +/- 404 | 3296 +/- 703 | 4130 +/- 476 |
|  | **dCry-RNAi** | 1941 +/- 269 | 3965 +/- 211 | 3439 +/- 441 | 2470 +/- 547 |  | 763  +/- 198 | 689  +/- 77 | 1089 +/- 166 | 1688 +/- 266 | 2431 +/- 374 | 2652  +/- 332 | 2851 +/- 301 | 2867 +/- 412 | 2632 +/- 551 |
| **ESA  (µm^2^)** | **24B-GAL4/+** | 525  +/- 109 | 851  +/- 153 | 1237 +/- 275 | 2369 +/- 245 | 1740 +/- 208 |  | 1484 +/- 165 | 2189 +/- 129 | 2602 +/- 239 | 2511 +/- 300 | 1741  +/- 296 | 1414 +/- 284 | 1080 +/- 504 | 1973 +/- 329 |
|  | **dCry-RNAi** | 601  +/- 42 | 661  +/- 80 | 1093 +/- 233 | 1321 +/- 444 |  | 754  +/- 199 | 685  +/- 77 | 1040 +/- 152 | 1451 +/- 207 | 1754 +/- 273 | 1649  +/- 142 | 1191 +/- 170 | 1082 +/- 177 | 918  +/- 282 |
| **EDD-horizontal**  **(µm)** | **24B-GAL4/+** | 58 +/- 4 | 90 +/- 4 | 71 +/- 7 | 74 +/- 4 | 57 +/- 4 |  | 52 +/- 4 | 61 +/- 2 | 64 +/- 3 | 65  +/- 4 | 64  +/- 4 | 70  +/- 4 | 62  +/- 7 | 76  +/- 5 |
|  | **dCry-RNAi** | 59  +/- 3 | 83  +/- 3 | 69  +/- 4 | 59  +/- 8 |  | 35  +/- 3 | 36  +/- 2 | 44  +/- 3 | 48  +/- 3 | 56  +/- 4 | 62  +/- 2 | 66  +/- 2 | 61  +/- 3 | 61  +/- 3 |
| **ESD-horizontal (µm)** | **24B-GAL4/+** | 28  +/- 3 | 32  +/- 3 | 38  +/- 5 | 60  +/- 4 | 52  +/- 4 |  | 51  +/- 3 | 60  +/- 2 | 59  +/- 3 | 55  +/- 3 | 46  +/- 4 | 39  +/- 4 | 30  +/- 7 | 46  +/- 5 |
|  | **dCry-RNAi** | 29  +/- 1 | 27  +/- 2 | 32  +/- 4 | 38  +/- 6 |  | 34  +/- 3 | 36  +/- 2 | 43  +/- 3 | 44  +/- 3 | 45  +/- 3 | 48  +/- 2 | 38  +/- 3 | 34  +/- 3 | 30  +/- 4 |
| **FS-horizontal  (%)** | **24B-GAL4/+** | 52  +/- 2 | 65  +/- 3 | 55  +/- 4 | 31  +/- 4 | 14  +/- 5 |  | 2  +/- 2 | 8  +/- 3 | 19  +/- 4 | 24  +/- 3 | 33  +/- 4 | 45  +/- 5 | 55  +/- 7 | 40  +/- 3 |
|  | **dCry-RNAi** | 50  +/- 2 | 68  +/- 2 | 61  +/- 3 | 32  +/- 10 |  | 5  +/-  3 | 1  +/- 1 | 9  +/- 4 | 22  +/- 5 | 23  +/- 4 | 28  +/- 3 | 43  +/- 5 | 49  +/- 5 | 53  +/- 6 |
| **EDD-vertical  (µm)** | **24B-GAL4/+** | 35  +/- 4 | 70  +/- 4 | 59  +/- 5 | 61  +/- 3 | 44  +/- 3 |  | 40  +/- 3 | 54  +/- 3 | 63  +/- 4 | 71  +/- 5 | 68  +/- 6 | 75  +/- 6 | 72  +/- 10 | 84  +/- 5 |
|  | **dCry-RNAi** | 43  +/- 3 | 70  +/- 3 | 63  +/- 5 | 51  +/- 7 |  | 25  +/- 3 | 26  +/- 2 | 33  +/- 3 | 45  +/- 5 | 61  +/- 6 | 62  +/- 5 | 66  +/- 5 | 67  +/- 7 | 61  +/- 10 |
| **ESD-vertical  (µm)** | **24B-GAL4/+** | 20  +/- 2 | 27  +/- 3 | 31  +/- 4 | 51  +/- 3 | 41  +/- 3 |  | 39  +/- 3 | 52  +/- 2 | 58  +/- 4 | 59  +/- 5 | 46  +/- 5 | 37  +/- 5 | 29  +/- 8 | 52  +/- 5 |
|  | **dCry-RNAi** | 24  +/- 1 | 23  +/- 2 | 31  +/- 4 | 34  +/- 7 |  | 24  +/- 3 | 26  +/- 2 | 32  +/- 2 | 41  +/- 4 | 49  +/- 5 | 46  +/- 3 | 35  +/- 4 | 35  +/- 5 | 32  +/- 7 |
| **FS- vertical  (%)** | **24B-GAL4/+** | 39  +/- 3 | 62  +/- 4 | 56  +/- 4 | 32  +/- 4 | 14  +/- 5 |  | 1  +/- 1 | 9  +/- 3 | 19  +/- 4 | 28  +/- 4 | 38  +/- 4 | 51  +/- 5 | 63  +/- 6 | 37  +/- 3 |
|  | **dCry-RNAi** | 41  +/- 3 | 66  +/- 3 | 57  +/- 4 | 33  +/- 10 |  | 4  +/- 2 | 1  +/- 1 | 7  +/- 3 | 24  +/- 5 | 25  +/- 4 | 29  +/- 4 | 46  +/- 5 | 53  +/- 5 | 52  +/- 6 |

***** n – Number of flies imaged in each group at different developmental stages
