# Supplementary material for: A Circadian Clock Gene, Cry, Affects Heart Morphogenesis and Function in Drosophila as Revealed by Optical Coherence Microscopy
Source: PLoS One. 2015 Sep 8;10(9):e0137236. doi: 10.1371/journal.pone.0137236 (PMC4565115; doi:10.1371/journal.pone.0137236)
Supplement: S2 Table — (DOCX) [file pone.0137236.s005.docx]

| **Stage** | **dCry** | **dCry fold change**  **relative to Embryo stage expression** | **dAct5C** | **dAct5C fold change**  **relative to Embryo stage expression** |
| --- | --- | --- | --- | --- |
| Embryo24 | 0.10 | 1 | 3217.81 | 1 |
| L1 | 0.077 | 0.76 | 2396.64 | 0.74 |
| L2 | 0.098 | 0.96 | 3138.26 | 0.98 |
| L3 | 1.30 | 13 | 3532.23 | 1.10 |
| P1 | 11.24 | 110 | 3342.96 | 1.04 |
| P2 | 15.69 | 154 | 3075.51 | 0.96 |
| P3 | 14.28 | 140 | 661.40 | 0.21 |
| P4 | 12.24 | 120 | 2595.87 | 0.81 |
| AD1 | 64.69 | 634 | 1011.33 | 0.31 |
