# Supplementary material for: A Circadian Clock Gene, Cry, Affects Heart Morphogenesis and Function in Drosophila as Revealed by Optical Coherence Microscopy
Source: PLoS One. 2015 Sep 8;10(9):e0137236. doi: 10.1371/journal.pone.0137236 (PMC4565115; doi:10.1371/journal.pone.0137236)
Supplement: S3 Table — (DOCX) [file pone.0137236.s006.docx]

| **Cardiac parameters** | **24B-GAL4/+** | **dCry-RNAi** |
| --- | --- | --- |
| **EDD-vertical (µm)** | 81 +/- 17 | 50 +/- 16 *** |
| **ESD-vertical (µm)** | 46 +/- 18 | 32 +/- 10** |
| **EDD-horizontal (µm)** | 79 +/- 18 | 61 +/- 12*** |
| **ESD-horizontal (µm)** | 43 +/- 17 | 35 +/- 8 * |
| **EDA (µm^2^)** | 4175 +/- 1555 | 2168 +/- 904*** |
| **ESA (µm^2^)** | 1683 +/- 1101 | 914 +/- 439** |
